# Association of remote imaging photoplethysmography and cutaneous perfusion in volunteers

**DOI:** 10.1038/s41598-020-73531-0

**Published:** 2020-10-05

**Authors:** Stefan Rasche, Robert Huhle, Erik Junghans, Marcelo Gama de Abreu, Yao Ling, Alexander Trumpp, Sebastian Zaunseder

**Affiliations:** 1 Klinik für Anästhesiologie und Intensivmedizin, Universitätsklinik Leipzig, Leipzig, Germany; 2 Pulmonary Engineering Group, Klinik für Anästhesiologie und Intensivmedizin, Universitätsklinik Dresden, Dresden, Germany; 3 Institut für Biomedizinische Technik, Fakultät Elektrotechnik und Informationstechnik, Technische Universität Dresden, Dresden, Germany; 4 Institut für Informationstechnik und Biomedizinische Technik, Fachhochschule Dortmund, Dortmund, Germany

**Keywords:** Medical research, Cardiovascular diseases

## Abstract

Remote imaging photoplethysmography (iPPG) senses the cardiac pulse in outer skin layers and is responsive to mean arterial pressure and pulse pressure in critically ill patients. Whether iPPG is sufficiently sensitive to monitor cutaneous perfusion is not known. This study aimed at determining the response of iPPG to changes in cutaneous perfusion measured by  Laser speckle imaging (LSI). Thirty-seven volunteers were engaged in a cognitive test known to evoke autonomic nervous activity and a Heat test. Simultaneous measurements of iPPG and LSI were taken at baseline and during cutaneous perfusion challenges. A perfusion index (PI) was calculated to assess iPPG signal strength. The response of iPPG to the challenges and its relation to LSI were determined. PI of iPPG significantly increased in response to autonomic nervous stimuli and to the Heat test by 5.8% (p = 0.005) and 11.1% (p < 0.001), respectively. PI was associated with LSI measures of cutaneous perfusion throughout experiments (p < 0.001). iPPG responses to study task correlated with those of LSI (r = 0.62, p < 0.001) and were comparable among subjects. iPPG is sensitive to autonomic nervous activity in volunteers and is closely associated with cutaneous perfusion.

## Introduction

Monitoring tissue perfusion is a pivotal concern in critically ill patients^1^. Besides the recently proposed vital microscopy of the sublingual circulation^2^, the cutaneous microcirculation is a useful “body window to the circulation” and may indicate global perfusion deficits early in critical illness by clinical signs like skin mottling or prolonged capillary refilling^3–6^. Remote imaging photoplethysmography (iPPG) is an emerging technique that allows to sense cardiovascular signals in the outer skin layers^7^. The absorption and reflection of light at the skin is altered by local hemodynamic incidents, most importantly the cardiac volume pulse transmitted into the cutaneous and subcutaneous tissue. Minute fluctuations of the reflected light carry certain physiological information like heart rate and respiratory rate and can be read by conventional cameras. The ability of iPPG to measure these parameters remotely and to detect atrial fibrillation, for example, has been proved in numerous clinical settings^8–11^.

iPPG also has been shown to be sensitive to haemodynamic conditions beyond heart rate. To an individual extent, its signal intensity instantly responds to changes of pulse pressure and mean arterial pressure^12,13^. Owing to the penetration depth of light, this specific behaviour is restricted to the haemodynamic background in the cutaneous microcirculatory network. iPPG thus selectively targets the peripheral cutaneous circulation. Of special interest is the two-dimensional high-resolution signal pattern of iPPG. It yields a perfusion intensity map rather than a single spot measurement allowing a deeper investigation of cutaneous perfusion characteristics like density, heterogeneity and spread.

The emergence of iPPG signals is however still far from clear^14,15^. A varying light absorption by haemoglobin due to the cutaneous blood volume pulse is considered the key mechanism^16^ and indicates the sensitivity of iPPG for cutaneous perfusion. Besides, ballistocardiographic optical surface phenomena^17^ and mechanisms that are not directly mediated by cutaneous blood volume are suggested^18^. The actual association of iPPG signals to the peripheral perfusion is therefore unknown.

In this study we investigated the relationship of the iPPG to cutaneous perfusion. To this end, we compared iPPG signals to laser speckle imaging (LSI), a reference measure of cutaneous perfusion, in volunteers exposed to various stress situations that evoke autonomic nervous activity.

## Methods

### Participants

The study protocol was approved by the Institutional Review Board of the TU Dresden (IRB00001473, EK168052013). Thirty-seven volunteers participated in the study (28.8 ± 5.2 years, 15 females). Written informed consent was obtained from all participants. Participants who are shown in pixelized photos have given their explicit permission. All experiments were performed in accordance with the applicable guidelines and regulations.

### Measurements

Baseline and experimental measurements comprised simultaneous recordings of iPPG video, Laser Speckle contrast imaging and reading of a reference photoplethysmogram (PPG, IR Plethysmograph MLT1020FC, ADInstruments, Dunedin, New Zealand) of two minutes duration. iPPG videos were recorded by an industrial-standard CMOS camera (UI-3370CP-C-HQ, IDS Imaging Development Systems GmbH Obersulm, Germany) at 25 frames per second, a resolution of 960 × 600 pixel and a 12 bit colour depth. Measurements were made under usual indoor fluorescent lighting. Videos were processed offline for iPPG analyses. The green color channel with a wavelength between 520 and 550 nm was evaluated. Laser speckle contrast images (LSI) for reference measurements were made by the moorFLPI-2 Laser Speckle Contrast Imager (Moor Instruments Ltd., Axminster, UK). The Laser speckle contrast imager employed a coherent light with a wavelength of 785 nm.

Participants were lying in supine position throughout the experiments. Cameras and laser speckle imager were positioned nearly perpendicularly to the ROI (region of interest) on the forehead. The recording distance (patient face to device) was approximately 40–45 cm for laser speckle and 70–80 cm for the cameras. This setup was chosen as tradeoff between recording a sufficient large area from almost equal angles (perpendicular) and doing the record at a close distance. Both, iPPG videos and LSI recorded the upper facial area including the forehead.

### Experimental procedure

The experimental protocol was designed to prompt a weak thermoregulatory and autonomic nervous activity bypassing major haemodynamic reactions. To this end, a modified Stroop colour-word test and a deliberate Heat test were applied.

**Figure 1:**
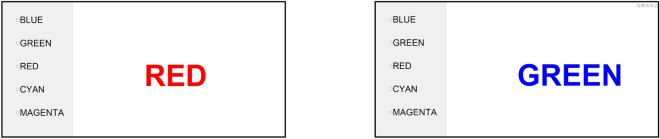
Representation of the congruent (left) and incongruent Stroop test with matching or different colour and meaning of the target word.

The Stroop colour-word test is a cognitive engagement test to trigger autonomic reactions^19^. Briefly, one of the colour words “MAGENTA”, “CYAN”, “BLUE”, “RED” or “GREEN” was displayed in the middle of a screen and participants had to quickly select the colour in which the word was printed from a list on the left side of the screen. Tests were performed as a congruent Stroop with matching colour and meaning of the target word as well as an incongruent Stroop where both did not match (Fig. 1). The display was mounted so that it was easy to see in a lying position. A computer mouse placed next to the participant was used for task interactions. The tasks were paced at a constant rate with a new colour word every 1.5 s. The Stroop test was carried out with customized software written in Matlab (Version 2018a, The MathWorks Inc., Natick, Massachusetts, USA).

The Heat test was done by wrapping supine subjects with a clinical temperature control blanket (HICO-AQUATHERM 660, Hirtz & Co. KG, Cologne, Germany) circulated by water pre-heated at 38 °C.

**Figure 2:**
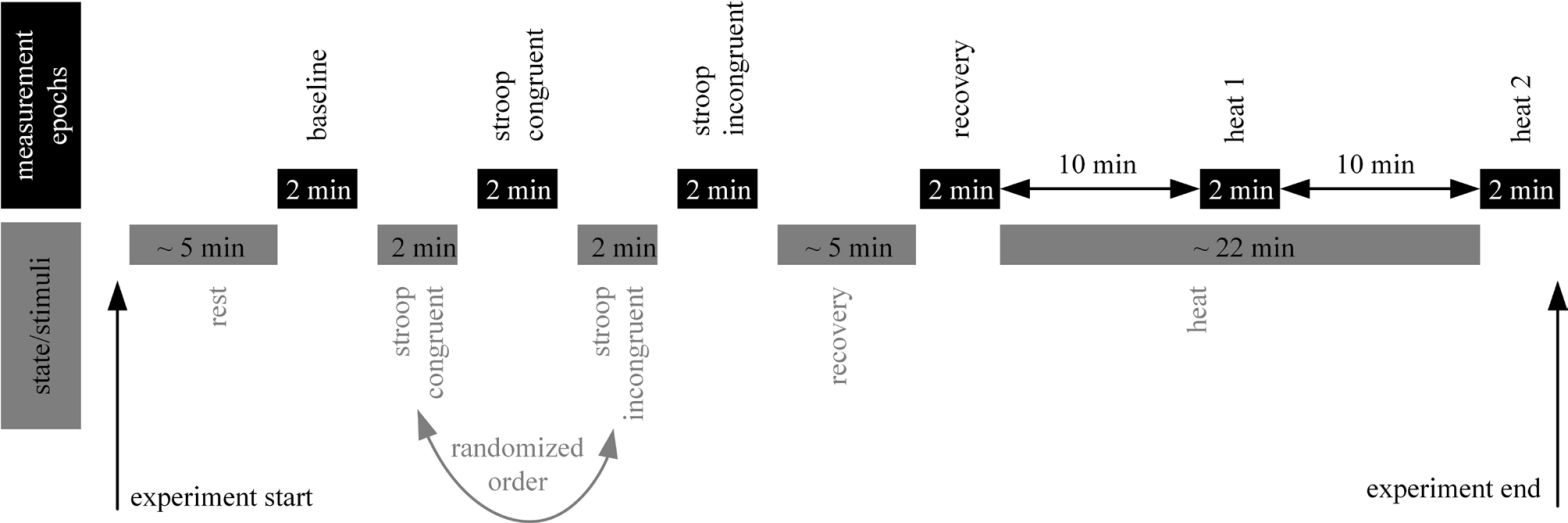
Outline of the study protocol.

After baseline recordings of iPPG-video, LSI and reference PPG, participants underwent the Stroop test. Congruent and incongruent tests were alternately sequenced per subject. Each part had a duration of two minutes. Immediately after each Stroop test, measurements were taken. Following a recovery period of five minutes a measurement was taken and subjects thereafter underwent the Heat test. Further measurements were done 10 min as well as 20 min after the beginning of the Heat test (Fig. 2).

### Data processing

#### ROI definition

**Figure 3:**
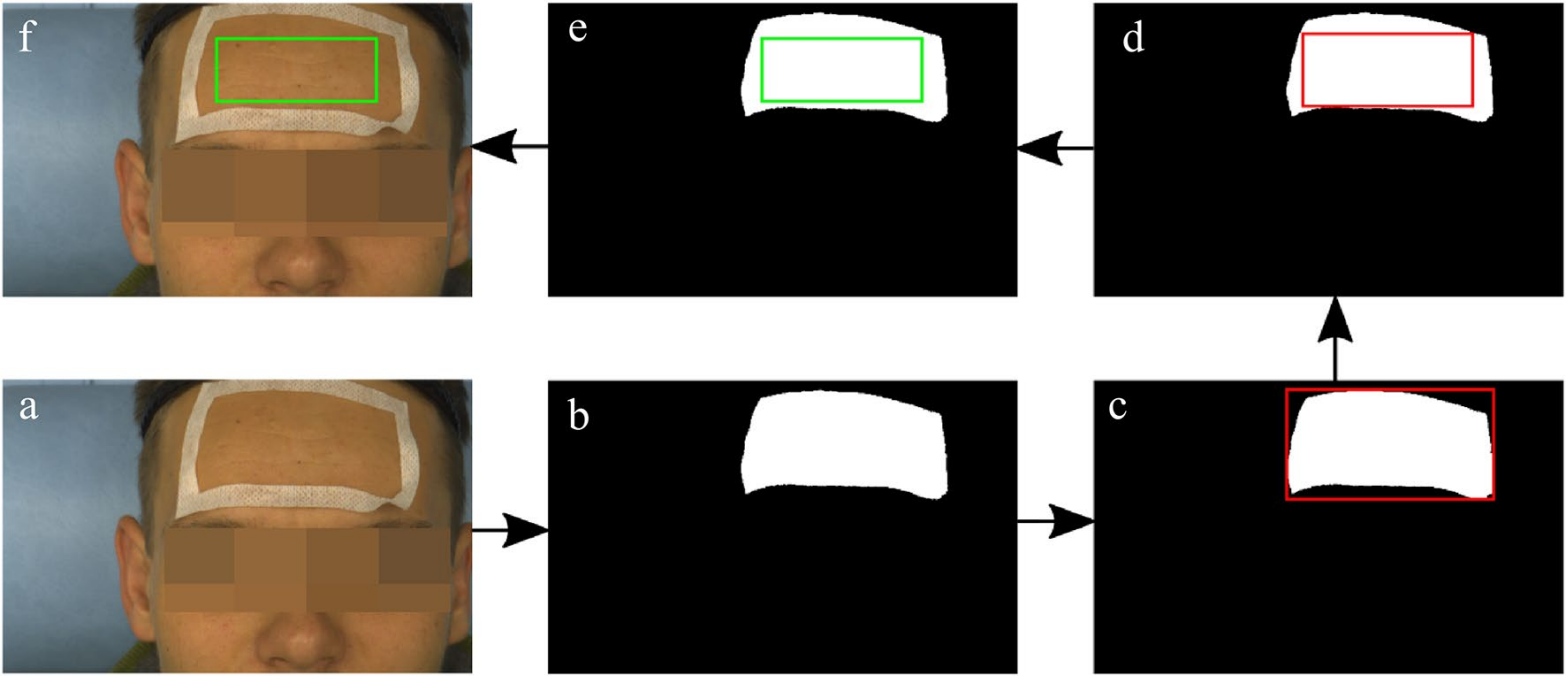
ROI definition algorithm. See text for details.

Our analysis focused on signals from the forehead because it is well perfused and has a large, homogeneous and plane area, which helps to avoid ballistocardiographic effects^15,20^. The same procedure to define the ROI was applied to iPPG and LSI recordings (Fig. 3). The ROI was kept statically throughout each measurement epoch. A tape on the subject forehead served as base for ROI definition (a). The area within the tape was identified (b) and the smallest circumscribing rectangle (c) was fitted. Through moving inward, the largest inscribing rectangle (d) was defined. This rectangle was reduced by 20 pixels in length and width (e) in order to account for slight body movements that otherwise might cause the tape to enter the static ROI (f) and introduce undesired effects. All ROIs were visually verified.

#### iPPG processing

For each measurement epoch, we analysed the iPPG video in an interval of 10 s duration starting 8 s after beginning of the respective measurement epoch. Afterwards, we averaged over the ROI to yield a time varying signal. A temporal linear interpolation was applied to construct an equidistant time series at a sampling frequency of 100 Hz.

iPPG was quantified in terms of a perfusion index $PI$ from each interval. $PI$ uses the iPPG signal’s AC and a DC component^18^ and was defined by $PI=\frac{A{C}_{iPPG}}{D{C}_{iPPG}}$. To calculate the DC component, $iPPG\left(t\right)$ was filtered by a Butterworth low-pass filter of 3rd order and cutoff frequency of 0.4 Hz to yield a DC signal $iPP{G}_{DC}\left(t\right)$. To define the AC component, another Butterworth low-pass filter was applied (5th order, cutoff frequency 8 Hz). The AC signal $iPP{G}_{AC}\left(t\right)$ resulted as difference between suchlike filtering and $iPP{G}_{DC}\left(t\right)$. Using $iPP{G}_{AC}\left(t\right)$, single heart beats were detected by the method proposed by Lázaro et al.^21^. Heart cycles were then defined around the time instants of detection using the median interval between all detections. Heart cycles showing a mean correlation lower than 0.3 in a pairwise comparison between all cycles were sorted out. From the remaining cycles, an average cycle was calculated. The average cycle was used to determine the AC amplitude as difference between its maximum and minimum value. The DC component was taken as average value of $cbPP{G}_{DC}\left(t\right)$ at the time instants of single beats.

#### Laser speckle processing and reference PI from finger PPG

Laser speckle processing and reference PPG used the same time intervals as iPPG. The procedure to define the ROI for LSI was identically (but using the video images from the device). As feature, the mean flux value (Flux) was used (mean value over the ROI and time interval). Laser speckle data thus did not require any preprocessing as the feature extraction was readily provided by the device. The reference finger PPG was evaluated in terms of the perfusion index like iPPG and denoted as reference-PI.

### Data analysis

Values of PI, Flux and reference-PI throughout the study are given as mean ± standard deviation in arbitrary units (au). Data were z-transformed at their grand mean ($x_{z} = \frac{x - \mu x}{{\sigma x}}$ , where *μx* denotes the mean value of x and* σx* its standard deviation) and then evaluated in mixed effects models (R Version 3.6.1.^22^) with individual subjects set as random effect. The responses of PI and Flux to the study interventions were defined as the difference to their respective baseline values. The variances of PI and Flux both at the individual level and between-subject level were estimated from the null model.

The effects of study interventions on PI were tested against baseline in a oneway analysis of variance.

The effects of Flux on PI at the respective study tests were estimated in an analysis of covariance including flux and study test. Random coefficient models were used to investigate the individual variability of flux effects on PI. The explanatory performance of Flux as a source of PI was estimated by its coefficient of determination (marginal *R*^2^)^23^. A proportional variance reduction at both, the individual and between subject level was calculated^24^.

The effects of the Flux response on the PI response were likewise evaluated in an analysis of covariance. Pearsson’s linear correlation for these test responses was calculated.

## Results

**Figure 4:**
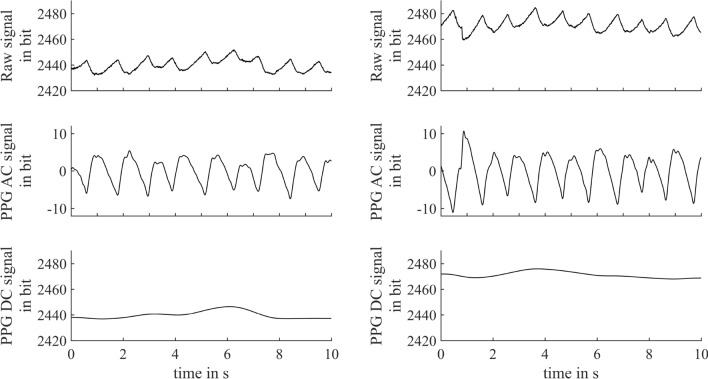
Exemplary imaging plethysmogram recorded at subjects´ forehead at rest (left) and after the Heat test (right). The top rows show the raw iPPG signal derived from light reflection in the green color channel, the middle the processed AC component and the bottom the DC component. An upward shift in the mean intensity and an increase in the AC component along with Heat test are clearly discernible.

All participants completed the experimental procedure. Recordings from six participants were discarded due to unexpected shutdowns of the iPPG camera or an inappropriate spatial synchronization between iPPG and LSI recordings. Representative plethysmograms derived from the iPPG video’s green channel of a subject at two different time points are shown in (Fig. 4). These plethysmograms were derived from the averaged pixel intensities over the ROI. A visual impression of the spatial perfusion mapping of iPPG is given as a supplementary file.

### Variance of PI and flux

Values of PI, Flux and reference-PI are given in Table 1. PI showed a marked heterogeneity between subjects compared to the individual level (90.8% of total variance between subjects versus 9.2% at individual level). Flux values were comparable in this regard (90.6% vs. 9.4%).

**Table 1:** Baseline values and effects of study interventions on iPPG’s Perfusion Index (PI), Laser Speckle Flux (Flux) and Reference PPG Perfusion Index (reference-PI).

	Baseline	Stroop congr	Stroop incongr	Recovery	Heat 1	Heat 2
PI	0.0055 ± 0.0016	0.0058* ± 0.0016	0.0059* ± 0.0019	0.0057 ± 0.0019	0.006* ± 0.0017	0.0061*± 0.0017
Flux	272.2 ± 60.7	276.2 ± 64.9	279.0 ± 70.1	277.2 ± 67.9	295.3* ± 81.7	297.7* ± 75.8
rPI	0.0034 ± 0.0018	0.0037 ± 0.002	0.0034 ± 0.0015	0.0034 ± 0.0016	0.0047* ± 0.0015	0.0049* ± 0.0015

Values are given as mean ± standard deviation. *p < 0.05 versus baseline.

### Effects of study interventions on PI

PI at individual subject level was systematically influenced by the study interventions (p < 0.001). PI significantly increased after both Stroop tests (congruent or incongruent) and during the Heat tests. Increases of PI ranged from 5.2% to 5.9% (Stroop tests) and from 10.1 to 11.8% (Heat tests) (Table 1). The test course (first or second Stroop) had a slightly stronger effect on PI than the test mode (congruent or incongruent Stroop) itself (model deviance 186.6 versus 188.8), suggesting a concomitant time effect. During recovery between Stroop and Heat tests, PI decreased to values not significantly different from baseline. Flux and reference-PI showed a weaker response and increased significantly only during the Heat test. The study interventions itself exerted no effects on heart rate. Heart rate significantly drooped during recovery after the Stroop test by 3.9 bpm (2.5–5.3 bpm, p < 0.001), but increased not beyond its initial level during the subsequent Heat tests (p 0.41–0.66 for the remaining measurements).

### Relation of PI to flux

Subjects’ individual PI significantly increased with higher Flux (*β* = 0.66, p < 0.001). In the analysis of covariance including Flux and study tests, these changes in PI were only determined by Flux. An independent effect of experimental tasks was not identified (p = 0.207). Apart from the recovery phase, where a slightly higher ratio of PI to Flux was found, the association of both variables was not affected by the study intervention. Despite the pronounced baseline variability of PI, the immediate effect of Flux on PI was comparable among subjects, a significant slope variance has not been proved (3.6% of total variance, p = 0.44). Flux explained a total of 45.3% of the variation of PI with a proportional variance change of 40.0% at subject level and 50.0% at inter-subject level.

### Responses of PI and flux

The response of PI to the study interventions was significantly associated to the Flux response (*β* = 0.56, p < 0.001). This relationship varied at the respective study interventions (p < 0.001 for the interaction with study intervention). While no significant association of the PI response to the Flux response was observed at the congruent Stroop test, this was found for the remaining experiments (Table 2). The relation of the PI response to the Flux response did not significantly vary between subjects (p = 0.79).

**Table 2:** Impact of the flux response on the PI response at the study tests.

	Estimate	CI	p
Overall association	0.56	0.43–0.68	P < 0.001
**Experimental tasks**			
Flux response, stroop congruent	0.06	− 0.29–0.40	0.747
Flux response, stroop incongruent	0.55	0.12–0.98	0.014
Flux response, recovery	1.11	0.68–1.54	< 0.001
Flux response, Heat 1	0.44	0.06–0.82	0.025
Flux response, Heat 2	0.52	0.13–0.91	0.010

Factor levels are compared by treatment contrasts against the Flux response at the congruent Stroop test. CI: 95% confidence interval.

**Figure 5:**
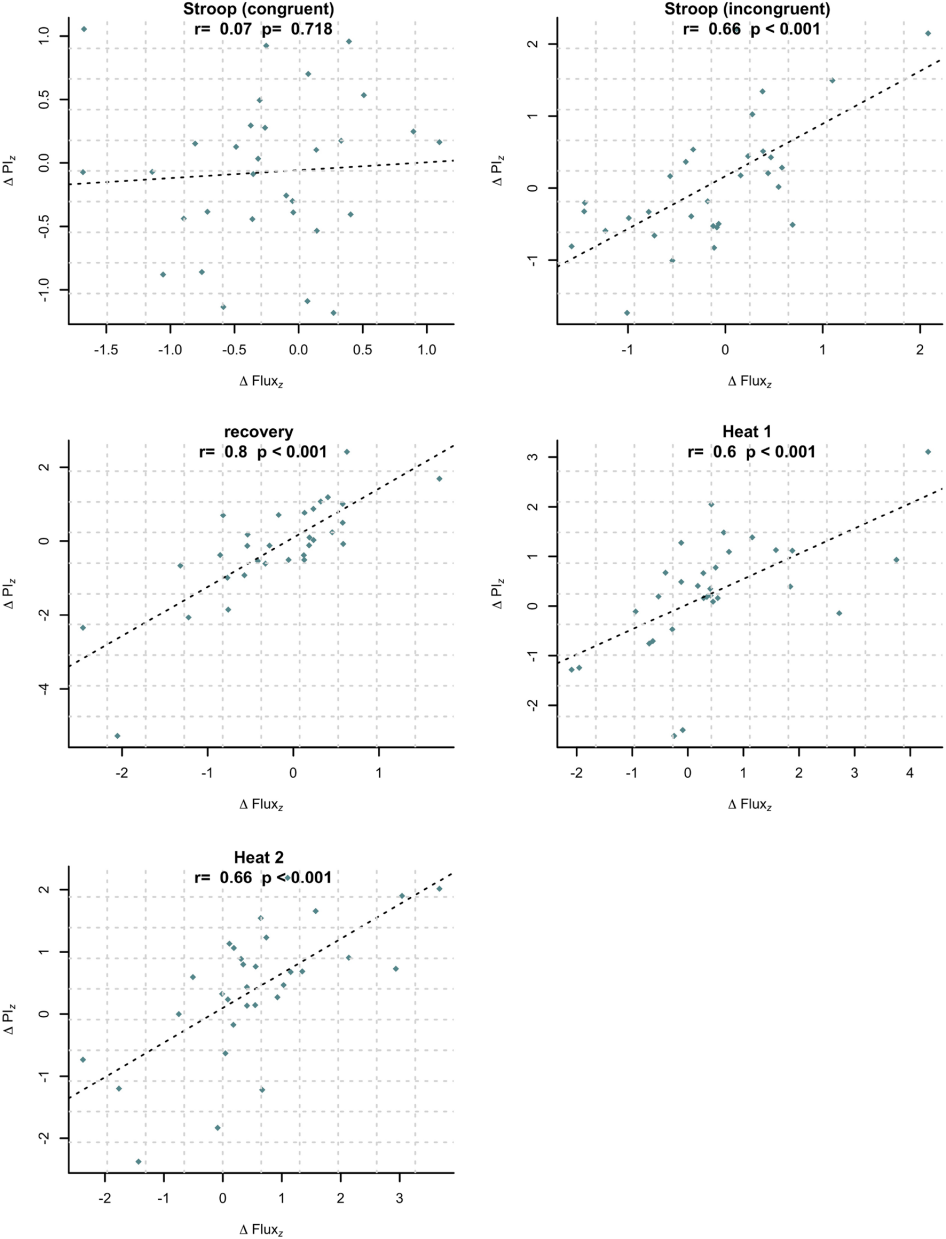
Correlation for PI response ${\Delta PI}_{Z}$ to Flux response ${\Delta Flux}_{Z}$ at each study intervention (values normalized).

Pearson’s linear correlation between the PI response and the Flux response ranged between 0.66 and 0.79 for the incongruent Stroop test, the recovery period and both heat tests (Fig. 5). At the congruent Stroop test, a significant correlation was not found.

## Discussion

The study results confirm that iPPG is determined by cutaneous perfusion in volunteers. A higher cutaneous perfusion measured by LSI was associated with a higher PI of iPPG. The variability of PI throughout the study was essentially explained by changes of perfusion. In addition, the experimental tasks systematically triggered significant changes of PI. PI changes followed those of LSI, i.e. both measures showed a corresponding response to stimuli.

Different factors determine iPPG. Cutaneous blood volume variations (BVV) following cardiac ejection are considered the key source of the plethysmographic signal detectable in the light reflection at the skin surface^7,12,25^. This relationship to cutaneous blood volume explains the dependence of iPPG on cutaneous perfusion.

The BVV effect is challenged by optical processes not directly linked to blood volume. The photoplethysmographic waveform is partly mediated by blood vessel wall movement, red blood cell (RBC) orientation and RBC aggregation/deaggregation^18,26^ without major volumetric alterations. In addition, the light absorption and scattering coefficients in superficial skin layers are affected by pulsation-induced compression of the cutaneous tissue, thereby prompting fluctuations in the light reflection at the skin surface and contributing to the iPPG waveform^14^. Another source of the pulsatile light reflection are ballistocardiographic optical phenomena (BCG) strictly occurring at the skin surface as a result of whole body micromovements and superficial tilting^17,27^, but without any optical interaction within the tissue.

The significance of BVV for the origin of iPPG is supported by physiological and experimental data. According to a cutaneous penetration depth of approximately 1 mm, iPPGs based on green light comprise local haemodynamics in capillary loops and the upper horizontal arteriovenous network of the papillary dermis^28,29^. Along with haemoglobin as the main light absorber in this spectral range in the dermis^28^, these features make up the functional basis for the plethysmographic representation of the cutaneous blood volume pulse. Although the pulsatile capillary pressure^30,31^ and velocity profile of erythrocytes^32^ are unlikely to exert a true blood volume effect in a single capillary due to the rigid nature of these vessels^32,33^, these phenomena underline the blood volume variation in their feeding vessels of the upper horizontal network. Whether the varying number of perfused capillaries per heartbeat essentially influences the iPPG remains to be investigated.

An intrinsic effect of the cutaneous haemoglobin amount on iPPG, and thus an association to cutaneous perfusion, is moreover emphasized by the peak signal strength of iPPG in the spectral range of the absorption maximum of haemoglobin^34^. The modulation of light absorption by haemoglobin along with BVV is therefore seen as a key effect. An adapted Lambert–Beer model was applied to explain this mechanism^35^.

LSI as a reference measure of cutaneous perfusion is based on a reduction of speckle contrast by the number as well as the velocity of moving blood cells and semiquantitatively assesses tissue blood flow^36^. Its optical background differs essentially from that of iPPG and in particular is not exposed to the effects of vessel wall movement, tissue compression or BCG. The significant relationship to LSI proved in this study therefore supports a predominance of cutaneous perfusion in the iPPG signal.

Recent studies on volunteers and patients affirm the association of iPPG to cutaneous perfusion. The local iPPG signal amplitude is significantly increased by the cutaneous vasomotor response to a circumscribed thermal impact^37^. Moreover, a cold face test in volunteers provoked marked changes in the iPPG signal even in entirely different body regions, indicating that systemic vasoactive events can be detected by iPPG^38^. Likewise, a capsaicin induced release of calcitonin gene-related peptide, known to be a potent vasodilator in the cutaneous microcirculation^39^, led to a more than two-fold increase of the iPPG signal amplitude in healthy adults and migraine patients^40^. Interestingly, iPPG was also found to indicate distinct migraine-associated blood flow changes^40^.

In a study comparing iPGG to Laser Doppler imaging (LDI) during local vasodilation after the application of a liniment or local heat, PI of iPPG and LDI-derived cutaneous blood flow multiplied almost identically and correlated closely^41^. It is to be noticed that the results of the current study confirm a concordance of iPPG with tissue perfusion even after a systemic and non-local vascular response of much lesser degree.

The presented study has some limitations. The far higher variance of PI among subjects, as compared to the intrinsic PI variance, has to be considered in signal analysis and evaluation. Its intraclass correlation and variance structure are comparable with those of the reference measure. Even with this parameter structure, a systematic variability of PI per subject in response to the study tasks was demonstrated. In parallel, the comparable response to Flux changes despite markedly different baseline levels of iPPG points to a consistent physiological basis of iPPG among subjects. This is an essential feature for iPPG as a monitoring device, especially in view of the intrinsically widely different PI values.

This study focused on the effects of peripheral vasodilation on iPPG. A significant response of iPPG to physiologically triggered vasoconstriction also has been demonstrated in volunteers^38^. However, further research is needed to clearly explain the behavior of iPPG during vasoconstriction, especially when it is associated with a decreased organ perfusion in critical disease.

The recording distance of approx. 40–45 cm between laser speckle and face slightly exceeds the recommended working distance of up to 38 cm. According to preliminary tests during experimental setup LSI did not show much differences in its applied configuration compared to a slightly closer application. Moreover, LSI meets the physiological expectation, suggesting that this distance has not compromised the results.

### Potential implications of the findings

iPPG might fulfill the requirements for assessment of peripheral perfusion in critically ill patients. iPPG has not yet been compared with clinical parameters of peripheral circulation, nor has it been thoroughly evaluated in patients with circulatory disorders. Its contribution to the detection of cutaneous perfusion deficits in the critically ill therefore remains to be investigated in dedicated clinical settings. The optimal iPPG parameters for the clinical estimation of perfusion deficits are subject to further research. Especially, the analysis of the two-dimensional dynamic perfusion maps opens up new perspectives for the evaluation of spatial perfusion parameters like density, heterogeneity and spread.

## Conclusion

iPPG is sensitive to autonomic nervous reactions in volunteers. iPPG’s signal intensity and its response to autonomic nervous reactions are closely explained by cutaneous perfusion.

## Supplementary information

Supplementary file1
