# Supplementary material for: Association of remote imaging photoplethysmography and cutaneous perfusion in volunteers
Source: Sci Rep. 2020 Oct 5;10:16464. doi: 10.1038/s41598-020-73531-0 (PMC7536393; doi:10.1038/s41598-020-73531-0)
Supplement: Supplementary file 1 — Supplementary file1 [file 41598_2020_73531_MOESM1_ESM.docx]

# Association of remote imaging photoplethysmography and cutaneous perfusion in volunteers

**Supplementary material**

Stefan Rasche (1)*, Robert Huhle (2), Erik Junghans (2), Marcelo Gama de Abreu (2), Yao Ling (3) Alexander Trumpp (3), Sebastian Zaunseder (4)

(1) Klinik für Anästhesiologie und Intensivmedizin, Universitätsklinik Leipzig, Germany

(2) Pulmonary Engineering Group, Klinik für Anästhesiologie und Intensivmedizin, Universitätsklinik Dresden, Germany

(3) Institut für Biomedizinische Technik, Fakultät Elektrotechnik und Informationstechnik, Technische Universität Dresden, Germany

(4) Institut für Informationstechnik und Biomedizinische Technik, Fachhochschule Dortmund, Germany

*Correspondence

Dr. Stefan Rasche

[st.rasche@me.com](mailto:st.rasche@me.com)

**Spatially perfusion mapping by iPPG and Laser Speckle Imaging**


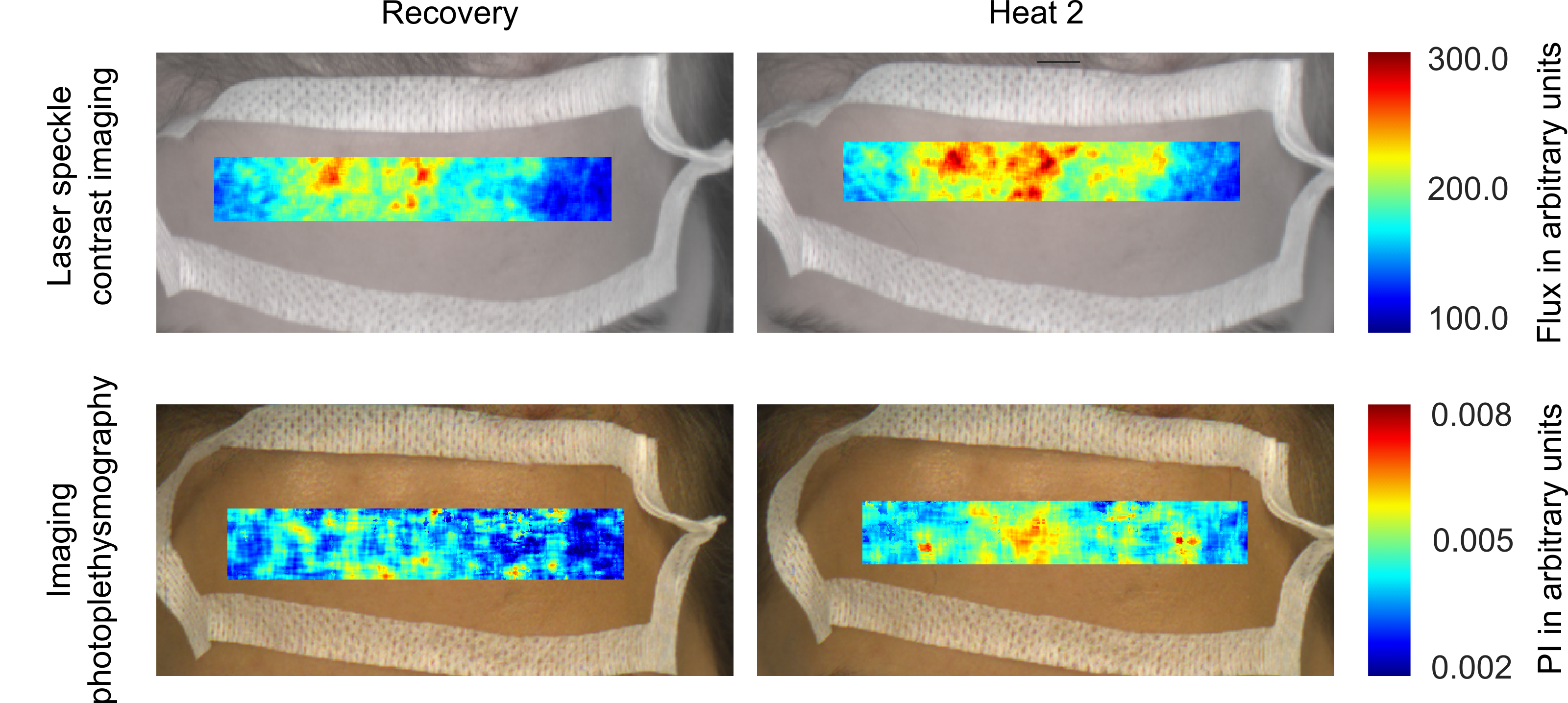


The visualization illustrates the two-dimensional, high-resolution pulse intensity pattern of iPPG signals along with its response to a whole body warming (Heat test).

To obtain the spatial visualization, recordings were processed (filtering and feature extraction) as described in the text but no averaging over the whole ROI was done. Instead, before filtering and feature extraction the recordings were spatially smoothed by an averaging filter of size 15x15 pixels.
